# αβ-T Cells Engineered to Express γδ-T Cell Receptors Can Kill Neuroblastoma Organoids Independent of MHC-I Expression

**DOI:** 10.3390/jpm11090923

**Published:** 2021-09-17

**Authors:** Josephine G. M. Strijker, Ronja Pscheid, Esther Drent, Jessica J. F. van der Hoek, Bianca Koopmans, Kimberley Ober, Sander R. van Hooff, Waleed M. Kholosy, Annelisa M. Cornel, Chris Coomans, Andrea Bisso, Marleen M. van Loenen, Jan J. Molenaar, Judith Wienke

**Affiliations:** 1 Princess Máxima Center for Pediatric Oncology, 3584 CS Utrecht, The Netherlands; J.G.M.strijker@prinsesmaximacentrum.nl (J.G.M.S.); J.J.F.vanderHoek-5@prinsesmaximacentrum.nl (J.J.F.v.d.H.); B.Koopmans@prinsesmaximacentrum.nl (B.K.); K.Ober@prinsesmaximacentrum.nl (K.O.); S.R.vanHooff-3@prinsesmaximacentrum.nl (S.R.v.H.); W.m.kholosy@prinsesmaximacentrum.nl (W.M.K.); or a.m.cornel@umcutrecht.nl (A.M.C.); j.j.molenaar@prinsesmaximacentrum.nl (J.J.M.); 2 Gadeta B.V., 3584 CM Utrecht, The Netherlands; r.pscheid@gadeta.nl (R.P.); e.drent@gadeta.nl (E.D.); chris.coomans@gadeta.nl (C.C.); a.bisso@gadeta.nl (A.B.); m.vanloenen@gadeta.nl (M.M.v.L.); 3 University Medical Center Utrecht, 3584 CX Utrecht, The Netherlands

**Keywords:** neuroblastoma, immunotherapy, γδ-T cells, TEG002, MHC-I

## Abstract

Currently ~50% of patients with a diagnosis of high-risk neuroblastoma will not survive due to relapsing or refractory disease. Recent innovations in immunotherapy for solid tumors are highly promising, but the low MHC-I expression of neuroblastoma represents a major challenge for T cell-mediated immunotherapy. Here, we propose a novel T cell-based immunotherapy approach for neuroblastoma, based on the use of TEG002, αβ-T cells engineered to express a defined γδ-T cell receptor, which can recognize and kill target cells independent of MHC-I. In a co-culture killing assay, we showed that 3 out of 6 neuroblastoma organoids could activate TEG002 as measured by IFNγ production. Transcriptional profiling showed this effect correlates with an increased activity of processes involved in interferon signaling and extracellular matrix organization. Analysis of the dynamics of organoid killing by TEG002 over time confirmed that organoids which induced TEG002 activation were efficiently killed independent of their MHC-I expression. Of note, efficacy of TEG002 treatment was superior to donor-matched untransduced αβ-T cells or endogenous γδ-T cells. Our data suggest that TEG002 may be a promising novel treatment option for a subset of neuroblastoma patients.

## 1. Introduction

In various adult cancers, innovations in immunotherapy, such as immune checkpoint inhibition, immunomodulatory antibodies, and engineered T cells expressing chimeric antigen receptors (CAR) have led to a big leap in survival rates [1]. For childhood solid cancers, however, immunotherapy is still in the early development stages, with one exception being the anti-GD2 antibody Dinutuximab for neuroblastoma patients [2]. Even though implementation of Dinutuximab into the neuroblastoma treatment protocol has significantly improved survival rates, the 5-year survival rate of ~50% for high-risk patients is still dismal and leaves room for improvement [3].

Neuroblastoma is a neuroendocrine solid tumor of early childhood. It represents 10% off all childhood cancers and ~15% of all childhood cancer related deaths [4]. Neuroblastoma generally presents as a solid tumor on the adrenal glands and/or on the sympathetic ganglia. The prognosis for survival of patients depends on several risk factors including age, level of metastases at diagnosis and MYCN amplification of the tumor [5,6]. Patients with low-risk disease have a favorable prognosis with >90% survival, while patients with high-risk disease have a chance of survival below 50% [4]. Since the chance of survival for high-risk patients has risen to ~50% after the addition of Dinutuximab to the treatment protocol, immunotherapy appears to be a promising approach for neuroblastoma. However, commonly studied immunotherapies engaging T cells have so far not shown overwhelming results for neuroblastoma, possibly due to the low or absent expression of MHC-I on neuroblastoma tumors [3]. Therefore, immune interventions which can eradicate tumor cells without the need of MHC-I would be a desirable approach. 

An innovative approach, which is independent of MHC-I, is the use of γδ-T cells. γδ-T cells are innate immune cells and represent 0.5–3.0% of circulating lymphocytes [7,8]. Most γδ-T cells express a T-cell receptor (TCR) that is composed of the variable regions Vγ9 and Vδ2. These TCRs can recognize tumor cells independently of the MHC-I complex, because rather than binding MHC molecules, Vγ9Vδ2 T-cell receptors recognize a conformational isoform of butyrophilin A1 (BTN3A1 or CD277) [9,10]. This isoform (also called CD277J) is expressed in the presence of elevated levels of phosphoantigens (IPP) that result from a dysregulated mevalonate pathway in stressed cells, such as tumor cells. IPPs are sensed by CD277’s intracellular domain and lead to a conformational change of its extracellular domain, which can be recognized by Vγ9Vδ2 T cells. CD277J expression can be triggered using aminobisphosphonates, such as pamidronate, that cause the accumulation of IPPs by blocking the farnesyl-PP-synthase, a crucial downstream enzyme in the mevalonate pathway [9,11]. 

Here, we propose to use this MHC-I independent tumor recognition capacity of γδ-T cells in the form of a novel cellular immunotherapy product called TEG002, as an immunotherapy candidate for neuroblastoma. TEG002 are αβ-T cells engineered to express the tumor specific Vγ9Vδ2 T cell receptor. They have a higher cytotoxic potential and can survive longer than endogenous γδ-T cells [12]. In this pilot project we have tested the potential efficacy of TEG002 therapy as a novel treatment for neuroblastoma, with patient-derived tumor organoids. We demonstrated that 50% of tested neuroblastoma organoids can effectively activate TEG002 and that killing of the organoids is independent of MHC-I expression. Hence, this pilot study identified TEG002 as a promising novel cellular product for immunotherapy for a subset of neuroblastoma tumors, warranting further investigations into its clinical application. 

## 2. Materials and Methods

### 2.1. Organoids and Culture Conditions

Tumor organoids were originally established from tumor material collected either at diagnosis, resection after several rounds of chemotherapy, or at relapse. The tumor was mechanically and enzymatically digested by cutting the sample in small pieces and treating with collagenases (I, II, and IV 10 mg/mL), respectively. After digestion, the tumor sample was placed in optimized culture medium: Dulbecco’s modified Eagle’s medium-GlutaMAX supplemented with 20% Ham’s F-12 Nutrient Mixture, 2% B-27 Supplement minus vitamin A, 1% N-2 Supplement, 100 IU/mL penicillin, 100 μg/mL streptomycin, IGF-1 (200 ng/mL), PDGF-AA (10 ng/mL), PDGF-BB (10 ng/mL), EGF (20 ng/mL), and FGF-2 (40 ng/mL). Tumor organoids were propagated at 37 °C in 5% CO_2_. On average, the medium was replaced every 3–4 days, and according to the size and confluence of the tumor organoids, they were passaged every 1–2 weeks.

### 2.2. Effector Cells and Culture Conditions

Effector cells were created from healthy donor peripheral blood T cells, at Gadeta BV. The TEG002 cells were engineered by transducing T cells with a defined Vγ9Vδ2-T cell receptor. The γδ TCR transductions in lymphocytes was previously described for the development of comparable product TEG001 [12,13,14,15]. TEG002 and TEG001 share the same functional moiety (i.e., the same receptor), and both display comparable anti-tumor reactivity. TEG002 are generated from αβ-selected T cells and prior to transduction T cells are activated by T Cell TransAct™ according to the manufacturer’s protocol (Miltenyi Biotec, Bergisch Gladbach, Germany). The entire manufacturing process of TEG002 is automated in GMP-compliant closed system.

Both the untransduced αβ-T cells and the endogenous Vγ9Vδ2-T cells from the same healthy donor were used as controls in all experiments. Effector cells were thawed 2–4 days prior to the addition to the target cells and rested in T cells medium containing human serum, penicillin/streptomycin, and IL-17 and IL-15. The Effector to Target (E:T) ratios for the co-cultures were calculated for the TEG002 and the untransduced αβ-T cells based on the transduction efficiency of the TEG002s (Supplementary Figure S1). 

### 2.3. Co-Culture Conditions

Tumor organoids were dissociated into single cells using both Accutase as an enzymatic method and pipetting up and down with a 20p tip on top of a 1000p tip as a mechanical dissociation method. The organoid cells were counted and plated in the optimal density for that organoid, which was obtained in previous growth curve experiments. This density differed from 10,000 until 40,000 single cells per well. Normal flat-bottomed 96-well plates were used. The cells were left in culture condition in 200 μL organoid medium for three days to form back into organoids before the effectors were added. On day three, the effectors were counted and added to the organoids in a 0.3:1, 1:1 and 3:1 E:T ratio in IMDM + 5% human serum + penicillin/streptomycin. As a lysis control, Puromycin (1 μg/mL) was added to the organoids. To enhance and/or trigger the accumulation of IPPs and stimulate TEG002’s recognition of the NBL tumor cells, each condition was repeated with addition of aminobiphosphanate pamidronate (10 μM). Three technical replicates were included for each condition.

For two organoids, the cytotoxicity and MHC-I independent recognition was tested in the presence of 10 μg/mL MHC-I blocking antibody (clone W6.32, Cat. NB100-64775, BioTechne, Minneapolis, MN, USA) or 10 μg/mL IgG isotype control (Cat. 31903, ThermoFisher, Waltham, MA, USA). The MHC-I blocking antibody was titrated in a co-culture with luciferase-transduced, MHC-I positive AMC691T organoids and HLA-restricted PRAME-TCR transduced T cells [16], which were kindly provided by our collaborators Dr. Stefan Nierkens and Annelisa Cornel. Cell viability was measured after 24 h of co-culture by adding luciferin and reading out the luminescence, which is emitted by the AMC691T cells through an ATP-luciferin reaction. 

### 2.4. Read-Out of Organoid Killing

After 24 h of co-culture, 50 μL supernatant of each well of the co-culture was collected and stored at −20 °C until IFNγ analysis using the Human IFNγ DuoSet ELISA (R&D Systems). ELISA was performed according to the manufacturer’s protocol. 

For four organoids, the cytotoxicity was further assessed, using the Live-Cell Imaging System Incucyte from Sartorius. Caspase3/7 Green (2.5 μM) was added to each well of the co-culture experiment to detect apoptosis, according to the manufacturers protocol [17]. The co-cultures were placed in the Incucyte system and pictures were taken of each well every 2 h for 72 h in total. 

### 2.5. Flow Cytometry

To assess the expression of CD277 and MHC-I on the organoids, Flow Cytometry experiments were performed. Naïve organoids, which were not co-cultured, were dissociated into single cells as described above. Cells were washed twice with cold FACS buffer (PBS + 2% FCS + 2 mM EDTA) and stained with a CD277-PE (1:20, Cat#130-117-693, Miltenyi Biotec, Bergisch Gladbach, Germany ) or HLA-a,b,c-PE (1:25, Cat#311406, BioLegend, San Diego, CA, USA) antibody for 30 min at 4 °C. Cells were washed twice again and prior to acquisition stained with DAPI (1:50) as a viability marker. Cells were stored at 4 °C until analysis using the Cytoflex LX cytometer. 

To test MHC-I expression on organoids in co-culture, cells were prepared in the same manner. After dissociation and washing, the samples were first stained with a Fixable Live Dead marker (eFluor 506, Cat. 65-0866-14, ThermoFisher, Waltham, MA, USA), then stained with a mix of CD3-AF700 (1:400, Cat. 300324, BioLegend, San Diego, CA, USA), HLA-a,b,c-PerCP (1:100, Cat. 311421, BioLegend, San Diego, CA, USA), CD56-APC (1:200, Cat. 318310, BioLegend, San Diego, CA, USA), and GD2-FITC (1:50, Cat. 563439, BD Biosciences, Franklin Lakes, NJ, USA) and measured on the Cytoflex LX.

### 2.6. RNA Isolation

For RNA isolation, the organoids were stored in TRIzol (Invitrogen) at −80 °C until use. On the day of isolation, the vials were thawed quickly at room temperature (RT). Then, 200 μL chloroform was added after which the vials were shaken vigorously for 15 s and left at RT for 2–3 min. Vials were then spun down for 18 min at 11,400 g at 4 °C. For all organoids, the Qiagen RNeasy Kit was used for the further preparation of the RNA sample, according to the manufacturer’s protocol [18]. RNA concentration was measured using a nanodrop. 

### 2.7. Whole Transcriptome Sequencing 

Paired-end libraries were prepared using the Illumina TruSeq Stranded mRNA kit and sequenced on an Illumina HiSeq 4000 (paired-end 100 bp, rapid mode). Sequencing reads were mapped to the 1000 Genomes phase 2 human reference assembly (NCBI build 37.1) using BWA (version 0.6.2) [19].

### 2.8. Data Analysis and Statistics

RNA sequencing counts were normalized to Transcripts Per Kilobase Million (TPM) per gene and log transformed. Differentially expressed genes between the cell populations were identified using the DESeq2 package in R 3.5.1 (CRAN), with input of raw read counts of all genes. Genes with false discovery rate (FDR) adjusted *p* value (padj) < 0.05 and log2 (Fold Change) > 1 or <−1 were considered differentially expressed. Principal component analysis (PCA) was performed in DESeq2 based on the constructed model. Pathway enrichment analysis was conducted in Gene Ontology/PANTHER publicly available online portal and REACTOME pathways with Bonferroni-corrected *p*-values < 0.05 were considered statistically significant [20]. For heatmap analysis, gene expression was mean-centered and scaled per gene and hierarchical clustering was performed with Ward’s method and Euclidian distance.

Incucyte data were analyzed using the Incucyte algorithm after which the RLU raw data were exported to Excel to analyze further. All data were normalized to the target only condition. Data were analyzed further using the Graphpad Prism8 software. FACS data were analyzed using the FlowJo software v10.7.1 as well as the GraphPad Prism8 software. 

## 3. Results

### 3.1. Expression of CD277 on Neuroblastoma Organoids

**Figure 1:**
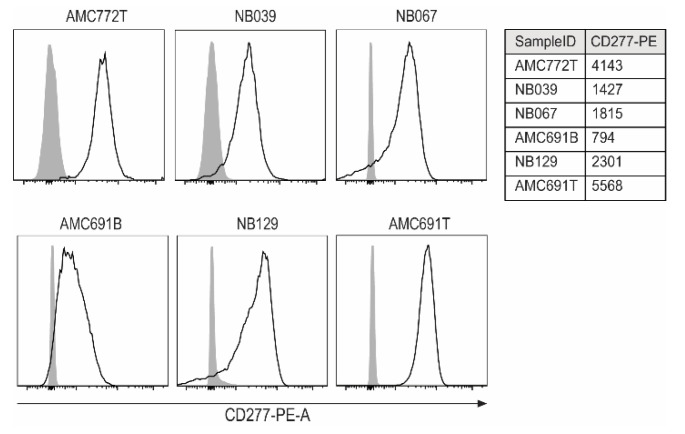
Expression of CD277 on neuroblastoma organoids. For each organoid, an unstained control served as a negative control (grey peaks). The table shows MFI of all samples for CD277PE.

To assess the feasibility of TEG002 therapy for neuroblastoma, we used six neuroblastoma tumor organoid models that were previously established from patient material [21]. We first wanted to know whether these organoids express CD277. Unfortunately, now no specific antibodies are available for the direct measurement of CD277J expression; however, CD277 expression on the organoids indicates potential for conformational change and recognition by TEG002 in the presence of high levels of IPP. All six organoids, albeit with different expression levels, express the TEG002 ligand CD277 (Figure 1). 

### 3.2. TEG002 Are Activated by 50% of Organoids in Co-Cultures

**Figure 2:**
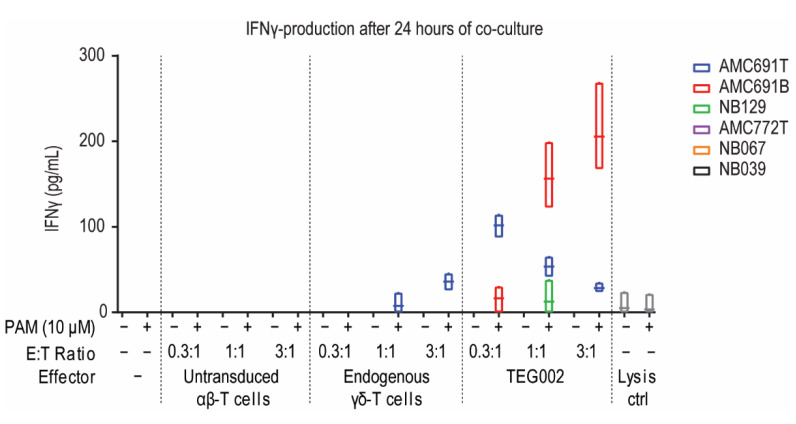
IFNγ concentration in supernatant of co-cultures reflecting effector activation. Supernatant of the co-cultures was taken at 24 h and measured by ELISA. Data are only shown when at least one of the data points was higher than 0. Grey for lysis control shows average of all organoids. Line in boxplot indicates the mean. Lysis control is puromycin (1 μg/mL). PAM = Pamidronate; E:T = Effector to Target; *n* = 3.

Since all organoids expressed CD277 and could thus potentially express the conformationally changed isoform serving as the TEG002 ligand, we initiated co-cultures to confirm the effector’s potency to recognize the tumor cells. The co-cultures were performed with TEG002, untransduced αβ-T cells, or endogenous γδ-T cells derived from the same healthy donor batch, to have controls with the same TCRs for the TEG002 product. IFNγ production by the effector cells, as measured in the supernatant after 24 h of co-culture, was used as a measure of effector activation upon organoid recognition. αβ-T cells and endogenous γδ-T cells did not show consistent activation upon exposure to neuroblastoma organoids (Figure 2): αβ-T cells did not produce IFNγ in any of the tested conditions, while endogenous γδ-T cell activation was detected only in co-cultures with AMC691T at high effector-target (E:T) ratios however, only in the presence of pamidronate that induces accumulation of IPP, which can lead to the conformational change of CD277. TEG002, on the other hand, produced IFNγ in co-cultures with AMC691T, AMC691B, and NB129, at different E:T ratios, also in a pamidronate dependent fashion. This indicates that TEG002 may be more proficient than untransduced αβ-T cells and endogenous γδ-T cells to become activated by neuroblastoma cells. The αβ-T cells and γδ-T cells as well as the TEG002s were not activated in the co-cultures with AMC772T, NB067, and NB039. In conclusion, TEG002 were superior to αβ-T cells and γδ-T cells from the same healthy donor and became activated in co-cultures with 3 out of 6 organoids. 

### 3.3. Neuroblastoma Organoids Susceptible to Recognition by TEG002 Have a Distinct Transcriptomic Profile

The differential susceptibility of organoids to recognition by TEG002 may depend on their functional characteristics. If so, understanding these features could provide insights into the mechanisms of tumor recognition by TEG0002 and could guide follow-up studies to optimize TEG002 efficacy for neuroblastoma. Moreover, determining the phenotypes of tumors could be an important step towards personalized treatment with TEG002, by identifying those patients with tumors susceptible to TEG002 recognition.

**Figure 3:**
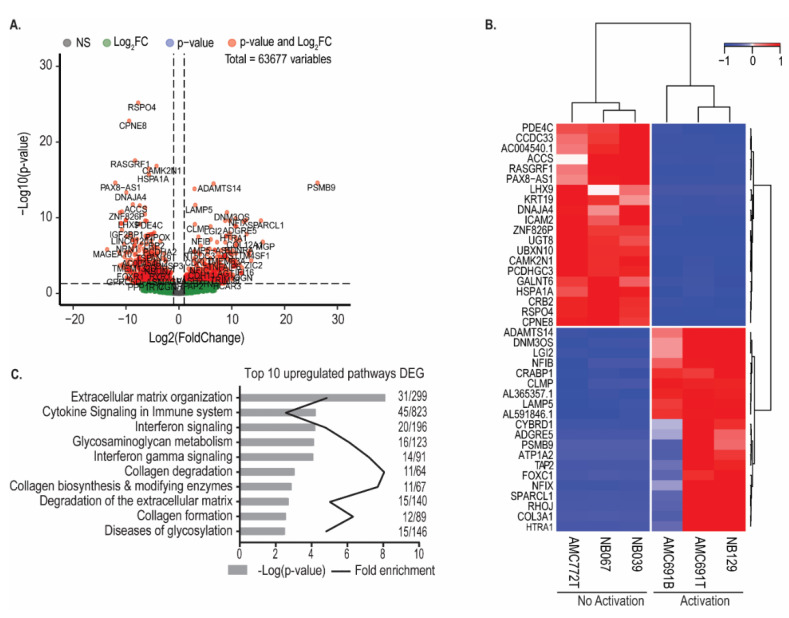
Organoids that activate TEG002 have a distinct transcriptomic profile. (**A**) Volcano plot of significantly differentially expressed genes between organoids that activated TEG002 and those that did not. (**B**) Heatmap of top 20 upregulated and top 20 downregulated genes. Expression values were scaled and centered. Clustering with Euclidian distance and Ward’s method. (**C**) Reactome pathway analysis of significantly upregulated genes in organoids that activated TEG002. Bonferroni-correct *p*-values are shown.

To investigate the functional characteristics of the organoids which could determine their susceptibility to TEG002, we divided the neuroblastoma organoids into two groups according to the IFNγ production by TEG002 (i.e., Activation versus No Activation) and compared their transcriptomic profiles. Of note, RNA profiling was performed on organoids under steady-state culture conditions, independent of the co-culture system. Differential gene expression analysis revealed 499 significantly upregulated genes (padj < 0.05 & Log2FC > 1) and 643 downregulated genes in organoids activating TEG002 compared to the 3 organoids that did not trigger TEG002 activation (Figure 3A). The heatmap in Figure 3B shows the expression of the top 20 upregulated and downregulated genes in the organoids that activated TEG002. Pathway analysis of the 499 significantly upregulated genes identified processes related to interferon signaling and extracellular matrix organization in organoids activating TEG002 (Figure 3C). Pathway analysis of significantly downregulated genes did not yield enriched pathways. 

### 3.4. Neuroblastoma Organoids Are Killed by TEG002 Independent of MHC-I Expression

**Figure 4:**
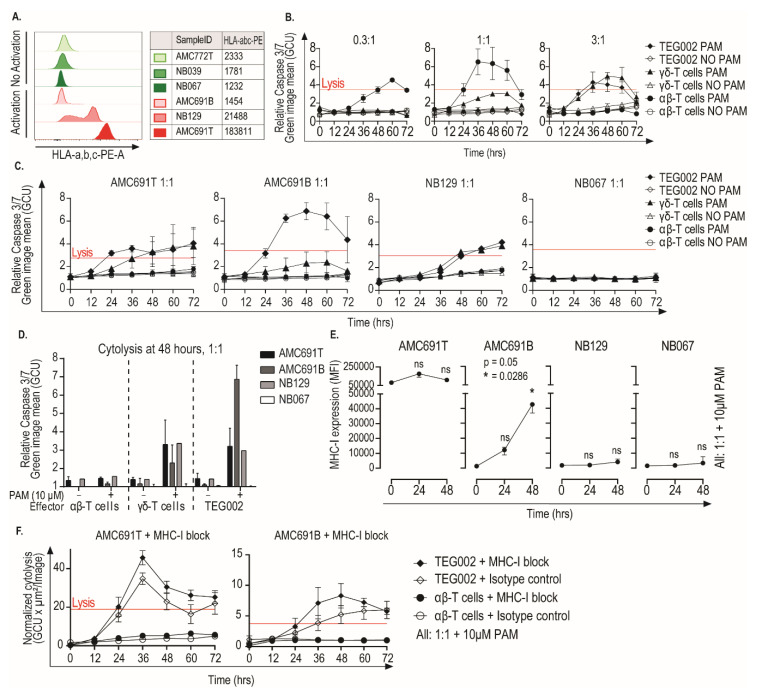
Neuroblastoma tumor organoids are killed by TEG002 in the presence of Pamidronate, independent of MHC-I expression. (**A**) Expression of HLA-a,b,c of all six organoids, measured by flow cytometry. Green indicates “No Activation” and red indicates “Activation” of TEG002. (**B**) Detailed dynamics of AMC691B killing by TEG002 over time. Representative graphs of one effector donor, which was tested in three different E:T ratios, are shown. Red line indicates the maximal cell lysis induced by the lysis control. (**C**) Killing of all four organoids by TEG002 over time at a 1:1 E:T ratio. AMC691T, AMC691B, and NB067 were tested with effectors from two different donors and NB129 with effectors from one donor. Graphs show mean of all experiments. (**D**) Bargraph of cytolysis at 48 h. Only the 1:1 E:T ratio is shown here. (**E**) Expression of HLA-a,b,c on four selected organoids in co-culture setting, measured by flow cytometry. Median Fluorescence Intensity (MFI) of HLA-a,b,c was used. Flow cytometry was performed only on organoids in co-culture with TEG002, with the addition of PAM at E:T 1:1. A Friedman test and a Dunn’s post hoc test, in comparison to T = 0, were performed to determine significant upregulation. (**F**) Cytolysis of AMC691T and AMC691B by TEG002 and untransduced αβ-T cells in the presence of 10μg/mL MHC-I blocking antibody (clone W6/32, Bio-Techne, supplementary Figure S3) or isotype control. Lysis control = Puromycin (1 μg/mL); PAM = Pamidronate; E:T = Effector to Target; all data are normalized to the target only condition; lysis control at 24 h.

We identified processes involved in immune activation, such as interferon signaling, as related to activation of TEG002. Since killing capacity of TEG002 is expected to be independent of MHC-I, we wanted to confirm this by investigating whether TEG002 activation by organoids resulted in effective killing of the tumor cells independent of the MHC-I expression of the tumor organoids. We measured MHC-I expression on the six neuroblastoma organoids (Figure 4A). Two organoids (NB129 and AMC691T) showed expression of MHC-I, both triggering TEG002 activation, while the other organoids did not display detectable MHC-I levels, including AMC691B, which did trigger TEG002 activation.

We assessed the dynamics and specificity of organoid killing by TEG002 over time in the three organoids that induced TEG002 activation (AMC691B, AMC691T, NB129). Since all organoids that were not recognized by TEG002 were MHC-I negative (Figure 4A), we selected one of those as a negative control for the killing assay (NB067). Organoid killing by effectors was assessed every 12 h, using a caspase-3/7-green assay.

Concordant with the observed IFNγ production (Figure 2), AMC691T, NB129, and AMC691B were killed by TEG002 in a pamidronate dependent fashion, and NB067’s viability was not affected (Figure 4B,C, Supplementary Figure S2 and Supplementary Videos S1–S4). A rapid cytolytic response of 12 h was observed in co-culture with AMC691T and AMC691B, while a longer co-culture of 36 h was required for cytolysis in NB129. The endogenous γδ-T cells also killed the organoids in a pamidronate dependent fashion, however, less effectively than TEG002 in the co-culture with AMC691B. Untransduced αβ-T cells were not able to kill the organoids. Notably, the MHC-I negative AMC691B organoid was killed by TEG002 more effectively than the MHC-I positive NB129 and AMC691T organoids (Figure 4D,E), supporting the fact that MHC-I expression is not required for recognition and killing by TEG002.

Activated TEG002 secrete IFNγ, which can cause, as previously described [22], an upregulation of MHC-I on neuroblastoma cells. This could imply that TEG002 directed killing could still be dependent of MHC-I if the neuroblastoma cells were to upregulate MHC-I during the co-culture. We therefore assessed the MHC-I expression of the organoids during co-culture. Both NB129 and NB067, which were respectively killed and not killed, did not show any significant upregulation of MHC-I during co-culture with TEG002 (Figure 4E). However, the organoid that was killed most efficiently, AMC691B, did show a significant upregulation of MHC-I, which coincided with elevated production of IFNγ (Supplementary Figure S3). We selected AMC691T, which naturally expresses MHC-I, and AMC691B, which significantly upregulates MHC-I, to further investigate the dependency on MHC-I for killing by TEG002. We co-cultured these two organoids with TEG002 in the presence of an MHC-I blocking antibody (Supplementary Figure S4). MHC-I blockade did not reduce the killing efficacy (Figure 4F). These results confirm that neuroblastoma recognition and killing by TEG002 is MHC-I independent.

## 4. Discussion

High-risk neuroblastoma is currently one of the deadliest cancers in small children. While innovations in treatment methods have increased the overall 5-year survival rate to ~50%, it remains unsatisfactory [4]. Neuroblastoma is an attractive candidate for immunotherapies, also considering the significant survival benefit achieved by implementation of Dinutuximab [2]. However, it remains a challenge that neuroblastoma tumors have several immune evasion strategies, including a low expression of MHC-I [3]. TEG002 is a novel cellular immunotherapy that uses the high cytotoxic potential of αβ-T cells together with the MHC-I independent tumor recognizing γδ-T cell receptor. Here, we have demonstrated that TEG002 are able to recognize and kill neuroblastoma organoids, even more potently than the effector donor matched αβ-T cells, which suggests a superiority of TEG002 over αβ-T cells. This indicates that TEG002 could be a promising immunotherapy for neuroblastoma. 

We showed that TEG002 can kill neuroblastoma organoids independent of their MHC-I expression, since even with an MHC-I blockade, TEG002 was still able to kill. This finding is in line with current literature on the recognition of tumor cells by γδ-T cells, which shows it depends on a conformational change of CD277 rather than the MHC-I complex [23,24,25]. Indeed, we observed that TEG002 were only able to kill neuroblastoma organoids when accumulation of phosphoantigens was induced using pamidronate, promoting the conformational change of CD277.

However, since not all organoids were killed, even in the presence of pamidronate, other underlying mechanisms are likely involved in the recognition and killing of the neuroblastoma organoids. Here, we have identified interferon signaling and extracellular matrix organization as processes which may support TEG002 activation by tumor cells. Remarkably, these processes were recently also identified by Lawson et al. [26] as important pathways in mediating the sensitivity of tumor cells to killing by cytotoxic T lymphocytes. This implies that these pathways may be universal processes determining the susceptibility of tumors to T cell mediated immunotherapy. 

For solid tumors in general and neuroblastoma in particular, the road to the clinic is still long for γδ-T cell-based therapies. A recent review article by Kabelitz et al. [27] discussed the two distinct approaches to current γδ-T cell-based clinical trials. The first approach, especially for patients with hematological malignancies, is to adoptively transfer autologous or allogenic ex vivo expanded γδ-T cells. This has resulted in some exceptional cases of partial remission and even complete remission, demonstrating the outstanding potential of endogenous γδ-T cell-based therapy [28]. The second approach activates γδ-T cells and expands them in vivo by administering aminobiphosphonates, such as zoledronic acid (ZOL), with or without IL-2 to patients. ZOL with a low dose of IL-2 induced a transient γδ-T cell activation in patients with for example lung cancer or renal cell carcinoma, but this had no clinical effect on tumors [27]. For patients with refractory neuroblastoma, treatment with ZOL and IL-2 indeed resulted in an increased amount of peripheral γδ-T cells [29], but unfortunately the expansion rate was still too low to be therapeutically effective. 

Hence, beside showing some encouraging proof-of-principle result, γδ-T cells-based therapy holds some intrinsic limitations that need to be overcome. In this regard, TEG002 aims at combining in one innovative product the tumor specific recognition mediated by Vγ9Vδ2-TCR with the high cytotoxic potential and the higher proliferation and survival characteristics proper of αβ-T cells, which makes TEG002 an alternative and more promising approach than endogenous γδ-T cells [15].

Another MHC-I independent immunotherapy option could be to use CAR-T cells. For neuroblastoma, there are some studies looking into CAR-T cell therapy targeting for example L1CAM or GD2; however, results remain unsatisfactory [30]. TEG002 have the unique benefit of using a tumor specific natural receptor which recognizes derailed intracellular metabolic processes, instead of extracellular expressed proteins, which makes TEG002 potentially more interesting than CAR-T cell therapies. Because TEG002 is more specific to tumor cells than CAR-T cell-based therapies, TEG002 is in potential safer than CAR-T cell therapies. However, this still needs to be proven in the context of neuroblastoma.

Our pilot study represents a proof-of-principle for the use for TEG002 immunotherapy in neuroblastoma. Our observations need to be further verified with the use of a higher number of samples and more model systems, including in vivo studies. Besides testing TEG002 efficacy on additional neuroblastoma organoids, the study of TEG002 effects on healthy tissues and organoids will be important to confirm our findings and investigate the possible off-target toxicity of TEG002 therapy. It has been shown previously that TEG001 does not affect healthy tissues, neither in vitro nor in vivo [12,14]. TEG002 and TEG001 share the same functional moiety (i.e., the same receptor), and both display comparable reactivity towards tumor and healthy cells. In contrast to TEG001, TEG002 is manufactured in a GMP-compliant closed system for automated production. Hence, the reactivity of TEG001 is expected to be identical to TEG002. In this regard, a Phase I clinical study has already been initiated to investigate the safety of TEG002 in humans (ClinicalTrials.gov Identifier: NCT04688853) and will hopefully pave the way for further clinical studies of TEG002, possibly including neuroblastoma.

Of note, our results showed that only 50% of the organoids could induce TEG002 activation, suggesting that other more complex features are affecting TEG002 efficacy. Consequently, TEG002 therapy will likely require patient selection before treatment. In this regard, we have generated novel insights into the susceptibility of tumor cells to TEG002 mediated killing by identifying signatures of pathways—such as interferon signaling and extracellular matrix organization—associated with differential TEG002 activation. These observations warrant further validation to understand whether these biological processes can functionally determine the susceptibility for TEG002 killing and might play a universal role in the susceptibility of tumors to immune interventions. Once validated, these signatures can be used as biomarkers to predict which tumors could be susceptible for TEG002 therapy and to guide patient selection in the clinical settings.

In conclusion, in this pilot study, we demonstrated that TEG002 can kill neuroblastoma organoids in a pamidronate dependent fashion, independent of the MHC-I expression of the organoids. This indicates that TEG002 therapy might be an interesting novel therapeutic option for neuroblastoma, as well as other for other solid tumors with a low expression of MHC-I.

## Data Availability

The data that support the findings of this study are available on request from the corresponding author, Judith Wienke.

## Supplementary Materials

The following data are available online at https://www.mdpi.com/article/10.3390/jpm11090923/s1. Figure S1: Transduction efficacy of TEG002. Figure S2: Cytotoxicity of effector cells on AMC691B, AMC691T, NB129, and NB067. Figure S3: IFNγ levels of repeated co-cultures. Figure S4: Titration of MHC-I blocking antibody. Video S1: Timelapse video of co-culture with AMC691B. Video S2: Timelapse video of co-culture with AMC691T. Video S3: Timelapse video of co-culture with NB129. Video S4: Timelapse video of co-culture with NB067.
