# Supplementary figures and images for: αβ-T Cells Engineered to Express γδ-T Cell Receptors Can Kill Neuroblastoma Organoids Independent of MHC-I Expression
Source: J Pers Med. 2021 Sep 17;11(9):923. doi: 10.3390/jpm11090923 (PMC8471928; doi:10.3390/jpm11090923)

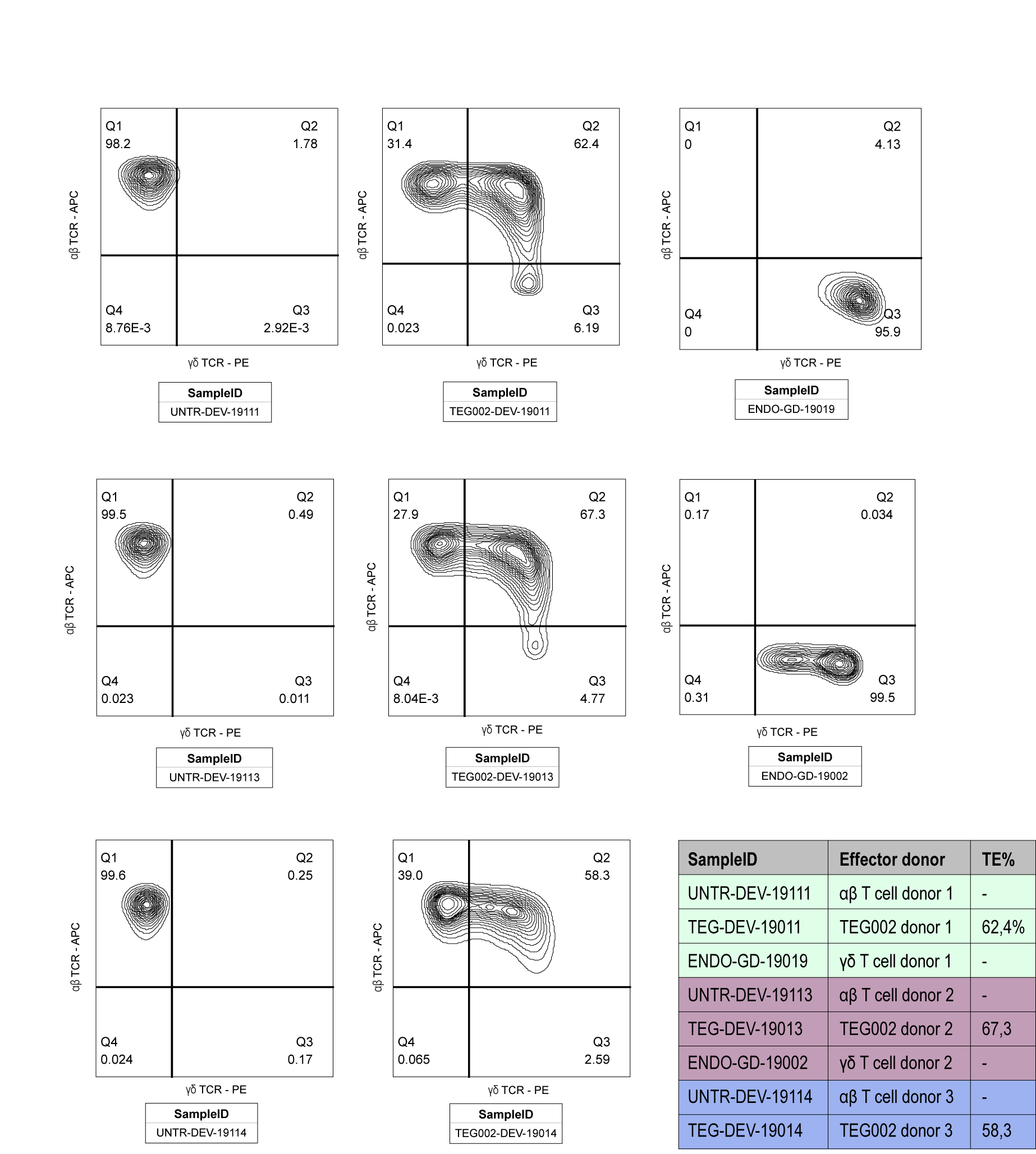

Supplement: Supplementary file 1 [file jpm-11-00923-s001.zip › 210903_Strijker et al_Supplementary 1.tif]

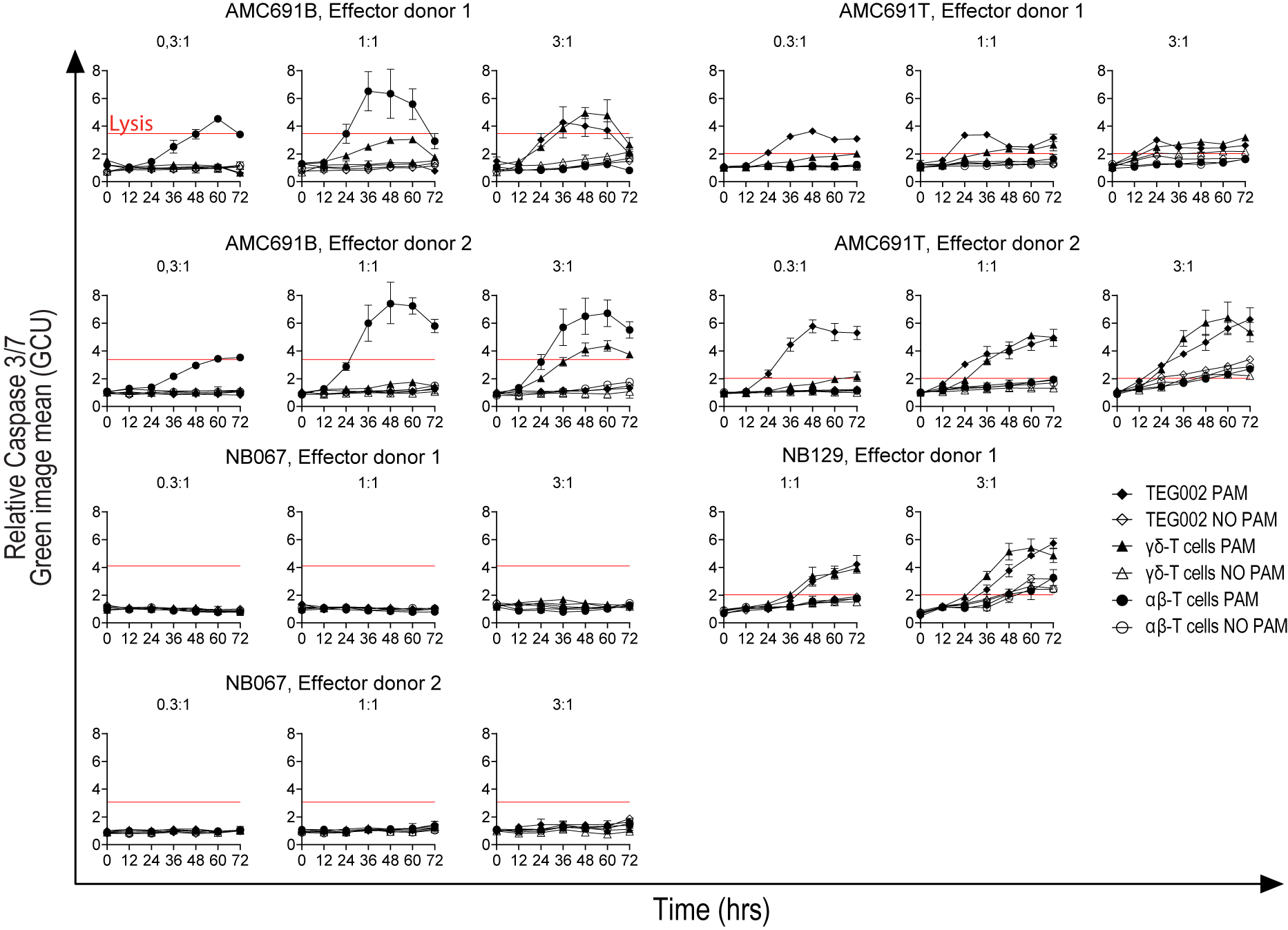

Supplement: Supplementary file 1 [file jpm-11-00923-s001.zip › 210903_Strijker et al_Supplementary 2.tif]

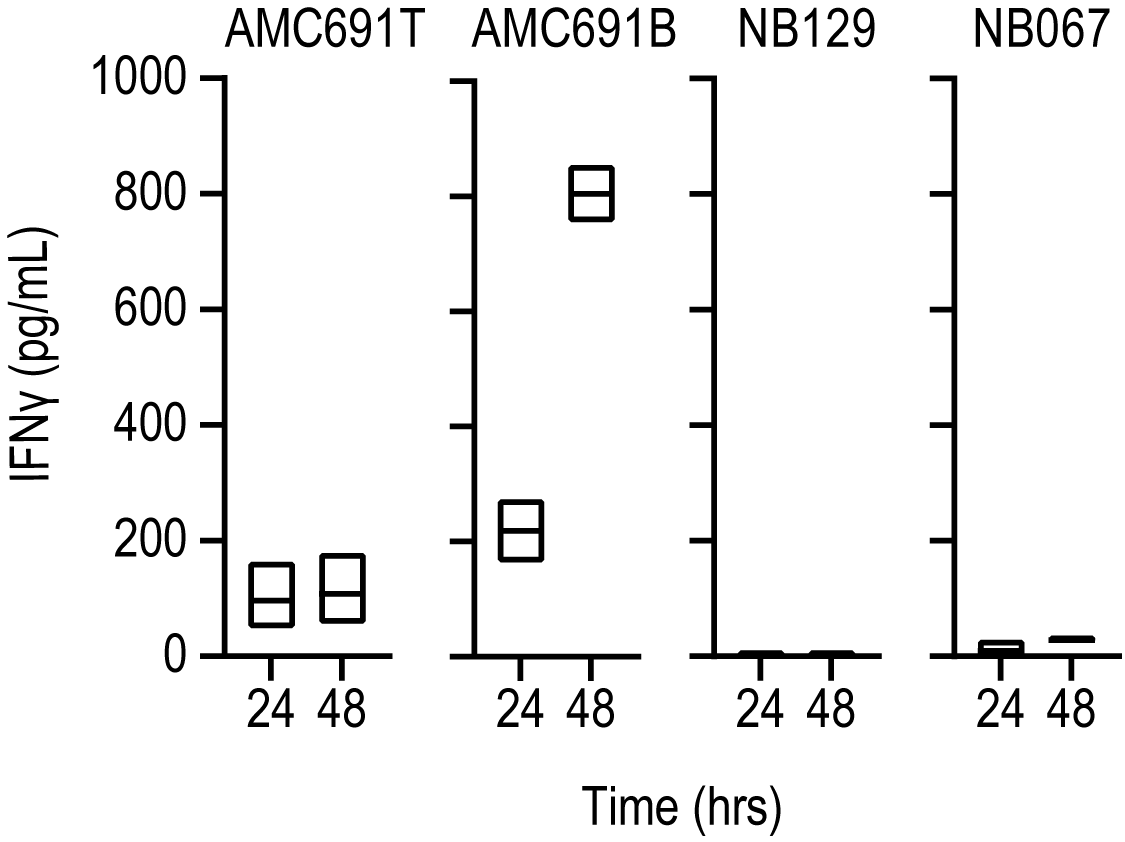

Supplement: Supplementary file 1 [file jpm-11-00923-s001.zip › 210903_Strijker et al_Supplementary 3.tif]

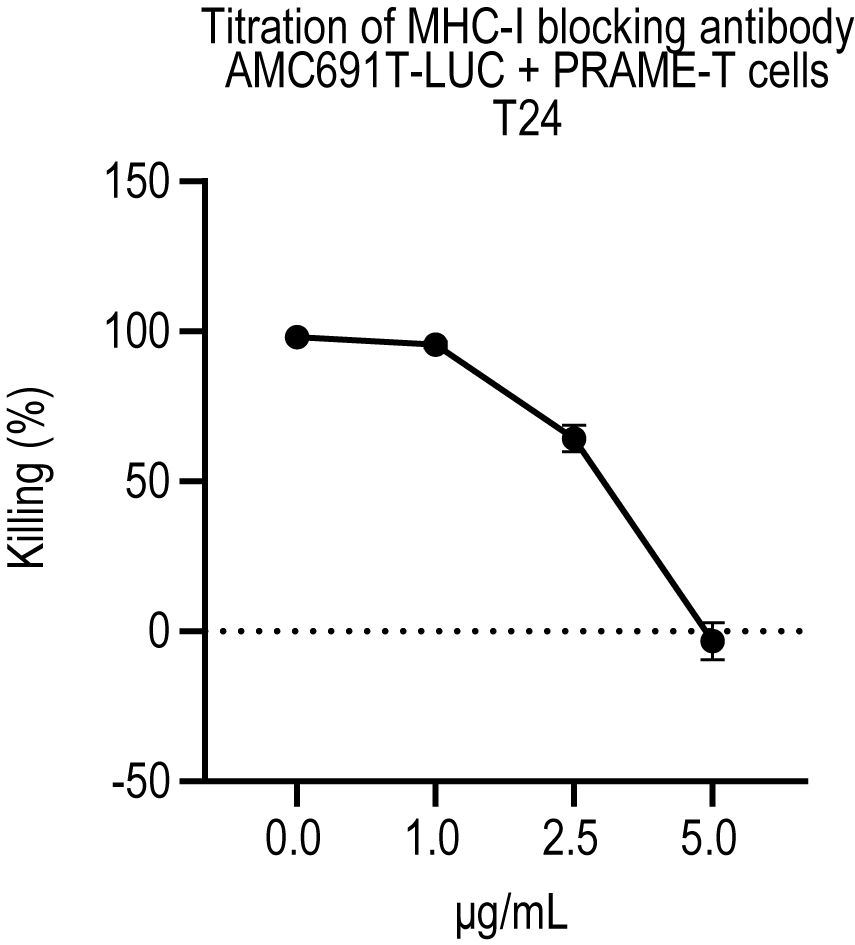

Supplement: Supplementary file 1 [file jpm-11-00923-s001.zip › 210903_Strijker et al_Supplementary 4.tif]
